# Associations of School-Level Factors and School Sport Facility Parameters with Overweight and Obesity among Children and Adolescents in Pakistan: An Empirical Cross-Sectional Study

**DOI:** 10.3390/sports12090235

**Published:** 2024-08-28

**Authors:** Moazzam Tanveer, Ejaz Asghar, Georgian Badicu, Umar Tanveer, Nadeem Roy, Asifa Zeba, Sameer Badri Al-Mhanna, Alexios Batrakoulis

**Affiliations:** 1 School of Physical Education and Sport Training, Shanghai University of Sport, Shanghai 200438, China; 2 Department of Allied Health Sciences, Health Services Academy, Islamabad 44000, Pakistan; 3 Department of Physical Education and Special Motricity, Transilvania University of Brasov, 500068 Brasov, Romania; 4 Department of Mass Communication, University of Lahore, Lahore 54000, Pakistan; 5 School of Physical Education, Shanxi University, Taiyuan 030006, China; 6 Department of Education, International Islamic University, Islamabad 44000, Pakistan; 7 Department of Physiology, School of Medical Sciences, Universiti Sains Malaysia, Kubang Kerian 16150, Malaysia; 8 Department of Physical Education and Sport Science, University of Thessaly, 42100 Trikala, Greece

**Keywords:** overweight and obesity, classroom exercise, sport facility, physical activity support, physical education activities, cross-sectional study

## Abstract

Childhood overweight and obesity are increasingly prevalent in Pakistan, posing significant public health challenges. This study explores the associations of school-level factors and school sports facility parameters with overweight and obesity among children and adolescents in Pakistan. A cross-sectional study across seven random districts in Punjab province, Pakistan, was conducted using a representative multistage random cluster sample. Underweight (BMI < 5th percentile), overweight (85th ≤ BMI < 95th percentile), and obese (95th percentile ≤ BMI) were defined using the US Center for Disease Control (CDC) 2000 criteria. Statistical analyses including the Chi-square test, Pearson correlation coefficient, and linear regression were performed to investigate predictive characteristics. Logistic regression analysis assessed the simultaneous impact of several covariates on dichotomous outcomes, with 95% confidence intervals (CIs) computed and a significance level set at *p* < 0.05. The study included 4108 Pakistani school children aged 9 to 17 years (mean age = 13.92 years, 59.3% boys) from 62 schools. The prevalence of overweight and obesity was 19.4% and 10.7%, respectively. Findings revealed a concerning lack of physical education activities (60% reported 0 sessions per week), morning exercise (60%), and classroom exercises (66%) among school-aged children. Leadership attitudes toward physical education (*β* = 0.04, *p* = 0.006) and students’ satisfaction with the playground (*β* = 0.05, *p* = 0.015) showed significant associations with body weight status. Conversely, provision of physical education facilities, effective fund utilization for physical education, meeting school sports facility requirements, and weekend opening of school sports grounds did not significantly impact weight status. Satisfaction with the playground was significantly associated with a lower risk of overweight (OR 0.81, 95% CI 0.69–0.95, *p* < 0.05), indicating reduced overweight likelihood among students satisfied with school playgrounds. The study underscores significant gaps in promoting physical activity within school environments and highlights the urgent need for interventions to enhance physical education resources and sports facilities, aiming to create healthier school environments and reduce overweight and obesity prevalence among students. Further research and interventions are crucial for promoting lifelong physical activity habits and improving student health and well-being.

## 1. Introduction

Physical activity is widely recognized as a critical health-promoting behavior for school-aged children and adolescents [1,2]. Extensive research demonstrates that regular physical activity during childhood and adolescence yields numerous benefits, including enhanced muscular strength and endurance, reduced risk of cardiovascular disease, maintenance of healthy weight, and improvement in physical and mental well-being [3,4]. However, physical activity participation is not solely influenced by individual factors but also by social and built environmental factors within schools and communities [5,6]. Hence, there is growing interest in identifying contextual factors or correlates that influence physical activity levels in these settings to inform the development of appropriate school- and after-school-based interventions aimed at increasing physical activity among children and adolescents [7,8,9]. Notably, regular physical activity plays a crucial role in preventing overweight and obesity among children, aligning with guidelines recommending at least 60 min of moderate-to-vigorous physical activity (MVPA) daily for children aged 2 to 17 [10]. Childhood overweight and obesity are persistent global health challenges associated with enduring negative health impacts [11,12,13]. The prevalence of overweight and obese children has risen substantially since 1970, affecting both high- and low-income countries [14,15]. This epidemic has significant short- and long-term repercussions on the physical, mental, and emotional health of children and their future as adults [16]. Examining the role of diet-related behaviors and habits in the development and prevention of overweight and obesity during childhood and adolescence is crucial, given that these behaviors are established early and can be challenging to modify later in life [17]. Addressing obesity and weight loss during adulthood becomes increasingly difficult, particularly after the age of 35 [18].

The school environment plays a crucial role in addressing childhood obesity at the socio-ecological school level. Schools have the potential to reach and engage millions of students over an extended period, making them a strategic setting for implementing cost-effective solutions and interventions [16]. With their captive audiences and institutional capacity, schools can consistently organize and implement interventions through their large staff of teachers and administrators [11,12]. However, it is important to note that schools alone may not effectively address the underlying issues that contribute to childhood obesity, as these factors often originate in the communities and households where children live [1,2]. While competitive food sales within schools are generally not directly associated with obesity, research suggests that efforts to reduce childhood obesity within schools may not always yield significant results [16]. However, schools still have the potential to support healthier eating habits and provide opportunities for physical activity [19]. The school environment, including the availability of physical education and opportunities for exercise on school grounds, the food environment within the school, and the social environment, can influence a student’s likelihood of being overweight or obese [20]. Schools are a vital component of childhood, particularly in countries like Pakistan, where a significant percentage of the population consists of children and adolescents [6,7,10]. With students attending school for several hours each day, for around 180 days a year, from ages five to seventeen, no other institution consistently interacts with a child in the same manner throughout their formative years [21]. Schools not only provide education but also serve as social environments where children interact with their peers and develop into young adults. Factors such as the availability of physical education programs, opportunities for physical activity on school grounds, the food environment within the school, and the social environment can all influence a student’s likelihood of being overweight or obese [22].

Obesity is a growing concern worldwide [13,14], and Pakistan, categorized as a low- and middle-income nation [17], has a sizable portion of its population—approximately 54%—falling between the ages of 0 and 19 years old [23]. Despite this youthful demographic, Pakistan faces significant challenges, grappling with the dual burdens of over-nutrition and poor nutrition, with around 50% of its population being overweight or obese, ranking it tenth among 188 countries [15,16]. Alarmingly, Pakistan has witnessed a steady increase in early fatalities associated with being overweight among both males and females over time [18,24]. Projections from the World Obesity Federation suggest that by 2030, 5.4 million Pakistani school-aged children will be obese [25,26]. Despite these concerning statistics, Pakistan has yet to implement operational policies to address overweight, obesity, and physical inactivity, as highlighted in the WHO Diabetes country profiles [17]. Although research on obesity among school-aged children and adolescents in Pakistan is limited, there is an urgent need for baseline data to assess its prevalence [25]. Childhood obesity is a global epidemic, affecting an estimated ten percent of school-aged children worldwide, with a quarter of them classified as obese [10]. Given the significant health risks associated with childhood overweight and obesity, including insulin resistance, hypertension, type 2 diabetes, and psychosocial issues, urgent prevention and control measures are imperative [13,17]. The school environment presents a promising platform for interventions, allowing for the regulation and modification of physical activity, food choices, and attendance patterns to effectively combat childhood obesity [8,9,10,11]. The existing literature on this topic is insufficient and may not provide an accurate representation of the current situation regarding overweight and obesity among school-aged children and adolescents in Pakistan [14]. Consequently, there is a need for comprehensive and up-to-date research that addresses the gaps in knowledge and understanding. To bridge this gap, it is essential to conduct population-based studies that utilize robust methodologies and incorporate diverse data sources [24,25,26,27,28]. This study should encompass a wide range of factors to capture the complexity of the issue. Moreover, it is crucial to employ standardized measurements and diagnostic criteria to ensure the comparability of findings across studies [15]. This will enable researchers and policymakers to develop a comprehensive understanding of the magnitude of the problem and its associated factors [29,30]. Efforts should also be made to update existing data and conduct regular surveillance to monitor changes in the prevalence of overweight and obesity among school-aged children and adolescents [31,32,33,34]. This will facilitate the development of evidence-based policies and interventions that are tailored to the specific needs of the population.

The objective of this study is to assess the prevalence of overweight and obesity among adolescents aged 9 to 17 years in Pakistan, utilizing a nationally representative sample. Furthermore, the study aims to investigate the association between school types, specific parameters related to school sports facilities, and the prevalence of overweight/obesity among children and adolescents in Pakistan. The ultimate goal is to provide evidence-based recommendations for preventing and managing overweight and obesity in these age groups. The findings of this study have broader implications for developing effective interventions, policies, and campaigns aimed at alleviating the burden of overweight and obesity among school-aged children and adolescents, not only in Pakistan but also in other regions worldwide.

## 2. Materials and Methods

### 2.1. Study Design, Setting, and Participants

In the summer of 2023, a population-based cross-sectional study was conducted among school-going children and adolescents aged 9 to 17 years in seven randomly selected districts of the Punjab region, Pakistan. A stratified multistage random cluster sampling method was employed to enroll a total of 4200 school-aged children and adolescents from 62 schools located in Lahore, Gujranwala, Gujrat, Sheikhupura, Narowal, Hafizabad, and Sialkot. Among the invited students, 4108 students (97.80%) completed useful questionnaires, while 92 students (2.2%) were disqualified due to insufficient information. Children aged 9–11 years old and adolescents aged 12–17 years old were sampled from grades 4 to 12, excluding grades 1 to 3 due to their inability to complete the study questionnaire [13]. Public schools were selected following approval from the Punjab School Education Department, which issued permission letters. Private school administrations independently granted permission upon visitation. The Punjab School Education Department (https://sis.punjab.gov.pk/, accessed on 23 December 2022) provided a list of schools from both urban and rural areas. A nominal fee of PKR 20 was charged in public schools, while private schools were charged PKR 10,000.00 to account for potential socioeconomic disparities. In instances where a school declined to participate, another institution was randomly selected [15]. The survey also involved collaboration from the Education and Rescue 1122 Departments, which volunteered to participate.

The Shanghai University of Sport Institutional Ethics Committee authorized the study (Approval Number: 1816111009-2022). Permission to conduct the study was obtained from the participating schools’ teachers and principals. Additionally, a letter of approval was received from the Punjab School Education Department. All participants provided voluntary written informed consent. The data were collected and processed anonymously.

### 2.2. Overweight and Obesity Status of Children and Adolescents

On pre-arranged dates, Rescue-1122 professionals visited the sampled schools to conduct anthropometric measurements of weight and height in the classroom. Data were collected directly from the students, ensuring the confidentiality of their responses throughout the process. Body weights were recorded to the nearest 0.1 kg, and heights to the nearest 0.5 cm [13]. BMIs were then calculated by dividing the person’s weight in kilograms by their height in meters squared (kg/m^2^). BMI classifications—underweight (BMI < 5th percentile), normal weight (5th percentile ≤ BMI < 85th percentile), overweight (85th percentile ≤ BMI < 95th percentile), and obese (BMI ≥ 95th percentile)—were determined based on age and gender-specific BMI percentiles according to the US Center for Disease Control (CDC) 2000 standards for children and adolescents aged 2 to 20 years [15,25,29].

### 2.3. Physical Activity and Physical Education

(1) School Sports Facility Parameters.

The school assessment focused on evaluating various aspects of school sports facilities and their impact on physical education. The questionnaire, written in English, was read aloud to students in lower grades [30]. Direct data collection from students was conducted with utmost confidentiality. A student self-reported questionnaire was used to assess the school sports facilities, and students responded to achieve the study’s objectives. The questionnaire used in this study was adapted from a previous study, incorporating items related to the following: (1) The attitude of school sports staff towards physical education in schools: (Reliability coefficient = 0.87) This item measures the attitude of the school sports staff towards the importance of physical education. It helps assess their support and commitment to promoting physical activity among students [30,35,36,37]; (2) The school provides students with access to sports facilities: (Reliability coefficient = 0.87) This item assesses whether the school offers sports facilities such as playgrounds, sports fields, or indoor sports facilities for students to engage in physical activities [36]; (3) Survey of children and adolescents’ satisfaction with playgrounds: This item measures the satisfaction levels of children and adolescents with the available playgrounds and sports facilities at the school. It helps understanding of whether the facilities meet the students’ expectations and needs [35]; (4) Physical education teachers’ availability in school: (Reliability coefficient = 0.74) This item evaluates the presence of qualified physical education teachers in the school. It assesses whether the school has dedicated professionals to teach physical education classes and guide students in physical activities [36] (5) The effective use of funds for physical education in schools: (Reliability coefficient = 0.84) This item explores how efficiently the school utilizes its allocated funds for physical education programs and activities. It assesses whether the funds are used to enhance the quality of physical education and promote students’ physical activity [35,36]; (6) The condition of the school’s sports equipment facilities to meet the requirements of daily physical education: (Reliability coefficient = 0.74) This item examines the state of the school’s sports equipment and facilities. It assesses whether the equipment and facilities are well maintained and sufficient to meet the needs of regular physical education classes [35,36]; (7) The school sports venue is open to students free of charge on weekends: This item assesses whether the school sports venue, such as the sports field, is accessible to students without any additional charges during weekends. It promotes opportunities for students to engage in physical activity outside of regular school hours. In the previous study, the test–retest reliabilities for these parameters were reported as 0.64 [35]. However, in this study, the internal consistency of the questionnaire, measured through Cronbach’s coefficient, was determined to be 0.79 for the seven items included. The design of the questionnaire was influenced by previous studies conducted by Wang et al. [35] and Kiyani et al. [36], which provided a format and structure for assessing school-level factors related to physical education and sports facilities.

(2) Situation of Fatness Tests at the School Level.

The assessment of fatness-related situations at the school level focused on several factors, including school sports facilities and their impact on physical education. The study utilized a self-reported questionnaire for sports teachers to assess the school fatness test-related situations. The questionnaire included the following items: 1. How many physical education (PE) classes in the last year were unable to start properly due to various factors such as weather conditions or overcrowding? (Reliability coefficient = 0.87). This item aimed to assess the extent to which external factors affected the implementation of physical education classes [36]; 2. What is the condition of the school sports equipment to support daily physical education activities? (Reliability coefficient = 0.74). This item examined the state of the sports equipment and facilities in the school and their adequacy to facilitate regular physical education classes [36]; 3. Is there a standardized procedure for evaluating students’ physical fitness? This item explored whether the school had established a consistent and standardized approach to assess the physical fitness of students; 4. What are the school’s statistics regarding the accuracy of evaluating the results of the most recent physical fitness examination? (Reliability coefficient = 0.84). This item sought to determine how effectively the school evaluated the outcomes of the physical fitness exams, ensuring accurate assessment and interpretation of the results [36]; 5. Does the school provide sports injury insurance for students? (Reliability coefficient = 0.78). This item assessed whether the school offered insurance coverage specifically for sports-related injuries to ensure the well-being and safety of students during physical activities [36]; 6. Are there any additional departments or organizations that support physical education in schools? (Reliability coefficient = 0.84). This item aimed to identify any external entities or departments that provided support, resources, or assistance in promoting physical education within the school [36]. In this study, the internal consistency of the questionnaire was measured using Cronbach’s alpha coefficient, with a value of 0.71. Previous study reliabilities, as measured by ICC, were 0.64 [35]. The questionnaire was adapted from a state-wide survey conducted by Wang et al. [35] and Kiyani et al. [36], providing a basis for the selection and formulation of relevant items for the assessment.

### 2.4. Statistical Analysis

Data analysis was performed using IBM SPSS Statistics version 26. Body weight status (BMI) was classified into four categories: underweight (BMI < 5th percentile), normal weight (5th percentile ≤ BMI < 85th percentile), overweight (85th percentile ≤ BMI < 95th percentile), and obese (BMI ≥ 95th percentile), based on the CDC 2000 BMI chart for children and adolescents aged 2 to 20 years [13,15]. For the prevalence analysis, a frequency distribution was generated. Bivariate analysis was conducted to compare body weight status (dependent variable) with school-level factors (independent variables) using the Chi-square test for trends [35]. The Pearson correlation coefficient (*r*) was employed to assess the strength of the correlation between independent variables and the body weight status. Linear regression analysis was used to evaluate the predictive power of school-level factors on body weight status. Logistic regression analysis was applied to estimate the simultaneous influence of multiple factors on the dichotomous outcome, with odds ratios (OR) and 95% confidence intervals calculated. Statistical significance was set at *p* < 0.05.

## 3. Results

Table 1 describes the participants of the study. The study comprised a total of 4108 participants, with 59.3% being boys and 40.7% girls. The majority of participants (96.2%) identified as Muslims, while 3.8% belonged to other religious groups. The sample encompassed both metropolitan (59.9%) and rural (40.1%) areas, ensuring diverse geographical representation. Regarding school types, 22.2% of participants attended private schools, while 77.8% attended public schools. Demographic characteristics and features of the selected schools are summarized. The research covered both urban and rural areas, with urban regions representing 53.2% of the sample and rural areas 46.8%. Public schools comprised the majority, constituting 72% of the sample, while private schools accounted for 27.4%. Regarding school levels, secondary schools made up the largest proportion at 61.3%, followed by higher secondary schools at 27.4%. Primary and middle schools constituted 6.5% and 4.8% of the sample, respectively. It is noteworthy that primary and middle schools are often linked to secondary or higher secondary schools, explaining the lower numbers in the study. Secondary and higher secondary levels of both public and private schools were combined for research purposes. This overview provides insight into the demographic composition and school-level distribution of the selected schools for the study.

**Figure 1:**
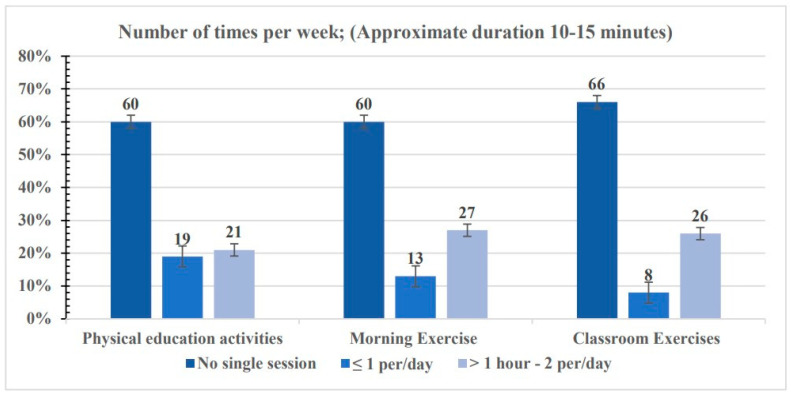
Descriptive statistics for the current prevalence of physical education activities, morning exercise, and classroom exercises number of times per week (approximate duration 10–15 min).

Figure 1 illustrates the current prevalence of physical education activities, morning exercise, and classroom exercises in schools, revealing that a substantial percentage of schools do not include these activities in their weekly routines. Specifically, the prevalence of 0 sessions per week for physical education activities is 60%, for morning exercise is also 60%, and for classroom exercises is the highest at 66%. These percentages highlight a significant gap in promoting physical activity and exercise within the school environment, suggesting the need for interventions to address this issue.

1. The Current Status of Fitness Test Implementation Across Educational Stages (Primary, Middle, Secondary, Higher Secondary) and Its Association with Different Types of Schools.

To compare the school type-specific prevalence of current fatness test-related situations at the school level, Table 2 presents notable findings regarding school-level environmental parameters across specific school types. The data indicate that 57.7% of public schools and 100% of private secondary schools reported reasons for failing to carry out physical education courses as planned over the past year. Poor fitness testing equipment was identified in 59% of public and 75% of private secondary schools. Additionally, 60% of public and 78.6% of private schools reported inaccuracies in students’ most recent physical fitness test data. Furthermore, 56.8% of public and 75% of private secondary schools lacked sports injury insurance for students. Other departments or agencies supported physical education in schools in 56.4% of public and 81.3% of private secondary schools. In private schools, one middle school, three secondary schools, and three higher secondary schools received such support, with a *p*-value of 0.030. The results also indicated that there were no sports or test-related facilities and parameters at the primary and middle levels among both public and private schools.

2. The Current Status of School Sports: Analyzing Facility Parameters and Their Association with Body Weight Status, with School Type-Specific Trends Among Children and Adolescents.

In Table 3, the comparison of prevalence of weight status with school-level parameters provides insights into the relationship between weight status and various school-level factors. Key findings reveal that a higher percentage of overweight and obese students, both from private schools (21.5% and 13.5%, respectively) and public schools (19.8% and 11.2%, respectively), reported poor behavior toward sports by school leaders. Similarly, concerning access to sports facilities, a higher percentage of overweight and obese students from both private and public schools expressed a lack of access (22.4% and 11.3% for private schools, 21.0% and 10.7% for public schools). Dissatisfaction with school playgrounds was prevalent among students from both types of schools, with significant percentages reporting dissatisfaction. Furthermore, a substantial proportion of students, regardless of school type, indicated a lack of physical education teachers and ineffective use of physical education funds. While body weight status showed no significant association with the majority of school-level sports facility parameters, addressing various school-level factors such as school leader behavior, access to sports facilities, school playground satisfaction, availability of physical education teachers, effective use of funds, provision of sports equipment, and opening of school grounds on weekends could contribute to promoting a healthier environment and potentially reducing the prevalence of overweight and obesity among students.

In Table 4, bivariate correlations between students’ body weight status and various school-level factors related to physical education and sports facilities are outlined. The findings are as follows: attitudes of leadership show a positive and significant correlation (*r* = 0.053 **) with students’ weight status, implying that as weight status increases, so do leadership attitudes within the school. Similarly, providing facilities indicates a positive and significant correlation (*r* = 0.049 **) with weight status, suggesting that as weight status increases, so does the availability of facilities within the school. Satisfaction with the playground demonstrates a positive and significant correlation (*r* = 0.065 **) with weight status, indicating that as body weight status increases, so does satisfaction with the school playground. However, there is no significant correlation (*r* = 0.021) between effective use of funds for physical education and weight status. Likewise, there is no significant correlation (*r* = 0.018) between school sports facilities and weight status. Finally, there is a positive and marginally significant correlation (*r* = 0.035) between school sports grounds open on weekends and body weight status, suggesting that as weight status increases, so does the likelihood of school sports grounds being open on weekends.

The linear regression analysis from Table 5 highlights the association between school-level parameters and students’ weight status. Among the six school sports facility parameters examined, two factors show significant effects on weight status: leadership attitudes toward physical education in schools (*β* = 0.04, *p* = 0.006) and students’ satisfaction with the playground (*β* = 0.05, *p* = 0.015). Conversely, providing facilities for physical education, effective use of funds for physical education, school sports facilities meeting requirements, and school sports grounds opening on weekends did not show significant associations with weight status. The overall analysis yielded an *R*^2^ value of 0.010, indicating a small portion of variance explained, with a statistically significant F-statistic (*F*(6, 3364) = 10.489, *p* < 0.001) for the regression model.

According to Table 6, logistic regression results show significant associations between school-level parameters and overweight and obesity. Key findings reveal that satisfaction with the playground was significantly associated with a lower risk of overweight (OR 0.81, 95% CI 0.69–0.95, *p* < 0.05), indicating a decreased likelihood of overweight among students satisfied with the school playground. Additionally, excellent leadership attitudes, providing sports facilities, and effective utilization of funds for physical education were linked to lower odds of overweight, though specific odds ratios and significance levels are not provided. For obesity, positive leadership attitudes, provision of facilities to students, and students’ satisfaction with the playground were associated with a lower risk (OR 0.73, 95% CI 0.588–0.91, *p* < 0.01; OR 0.76, 95% CI 0.61–0.95, *p* < 0.05; OR 0.71, 95% CI 0.58–0.87, *p* < 0.01, respectively). Similarly, effective funds for physical education and availability of daily physical education facilities and open sports grounds on weekends were associated with lower odds of obesity, albeit not statistically significantly.

## 4. Discussion

The objective of this study was to examine the current status of school sports, analyzing facility parameters and their association with body weight status, with school type-specific trends among children and adolescents in Pakistan. The results highlight significant deficiencies in the promotion of physical activity and exercise within school settings, including a notable lack of physical education programs, morning exercises, and classroom activities. These findings point to challenges across various school types and emphasize the need for enhanced physical education resources, standardized assessment processes, and sports injury insurance and support. The comparison of body weight status with school-level parameters underscores the importance of factors such as school leadership behavior and access to sports facilities in fostering a healthier environment and potentially reducing rates of overweight and obesity. The study indicates that leadership attitudes and student satisfaction with playground facilities play a crucial role in influencing body weight status. Effective allocation of funds for physical education and the quality of playground facilities are key factors in mitigating the risk of overweight and obesity.

Current estimates indicate that 19.4% of Pakistani school-aged children and adolescents are overweight, while 10.7% are obese. Studies conducted in Pakistan have reported varying rates of overweight and obesity among different age groups. For example, a study in Lahore found that 17% of primary school children aged 5 to 12 were overweight, and 7.5% were obese [15]. In the Hyderabad urban region in 2013, 12% of students in grades 6 to 10 were obese, and 8% were overweight [38]. Another study in Karachi reported that 19.1% of school children aged 11 to 15 years were overweight, and 10.8% were obese [25]. Additionally, a local survey in Lahore showed that 11.9% of students in private schools in grades 6 and 7 were obese, with 21.8% being overweight [39]. In Multan, a 2018 study on children aged 3 to 18 years revealed that 10% were overweight and 5% were obese [40]. According to the World Obesity Federation’s 2018 estimates, 6.6% of Pakistani children were obese, and 10.7% were overweight [41]. The current study indicates a higher prevalence of overweight and obesity among children and adolescents in Pakistan compared to previous studies.

The school environment plays a significant role in shaping a student’s life, and our study examined various aspects of school behavior, considering both public and private school settings. We found a significant association between school type and overweight and obesity among students, which aligns with the findings of previous studies [6,19,20]. Exploring different educational levels, our study revealed variations in student attitudes and behaviors. Specifically, our findings indicated that elementary school students were more likely to be underweight compared to other educational levels. On the other hand, secondary and higher secondary students exhibited different patterns in terms of attitudes and sleep patterns. Senior students were more prone to being short or long sleepers and spending more than two hours daily watching television, which is consistent with earlier research [13,14]. In Pakistan, this study identified a significant correlation between educational level, school type, and weight status, which aligns with the findings of previous studies. Secondary school students had a higher prevalence of obesity, while high schools had a higher incidence of overweight. Primary schools, on the other hand, had a higher proportion of underweight students [10]. Additionally, private schools had a greater number of overweight and obese students compared to public schools [6,21]. Considering that children spend a significant amount of time in the school setting during their childhood and adolescence, schools have an important role to play in promoting and supporting healthy habits among students. Creating a safe and supportive environment in schools is crucial, as it has been found that not attending a school perceived as safe increases the chances of children being overweight or obese.

Furthermore, engagement in physical activity and participation in physical education programs were found to be significant predictors of a child’s weight status, including being underweight, overweight, or obese [42,43,44,45]. These findings highlight the importance of fostering a positive school environment that promotes student engagement, physical activity, and academic success as part of comprehensive efforts to address childhood overweight and obesity [23]. By addressing these factors at the school level, interventions can be designed to create a supportive and healthy environment that positively impacts students’ weight status and overall well-being. The insights provided by this study regarding the relationship between school-related factors and childhood overweight and obesity are valuable [10,19,20]. It was observed that students who displayed little interest or enthusiasm for learning had a higher risk of becoming overweight or obese, emphasizing the need for educational environments that foster motivation and engagement. The perspectives of school officials on sports were found to have a significant impact on children’s health. This highlights the role of schools in promoting physical activity and sports participation, which can contribute to better weight outcomes among students. Additionally, it underscores the importance of educational opportunities in promoting overall health and healthy lifestyle behaviors among children [6,10,20]. The study also highlighted detrimental effects on students’ academic motivation, which can have implications for their risk of overweight and obesity. Creating a safe and supportive school environment is crucial not only for academic achievement but also for promoting the well-being of students and reducing the risk of overweight and obesity [46,47]. School absences were identified as a reflection of underlying stressors in students’ lives, which can impact their physical and mental health. Recognizing and addressing these stressors is important for supporting students’ well-being and reducing the risk of overweight and obesity [47].

The presence of regular physical education programs and dedicated physical education teachers were associated with a lower prevalence of weight problems among students. This highlights the importance of providing opportunities for physical activity within schools and the role of supportive school environments in promoting students’ overall well-being [44,45,46]. Importantly, the study emphasized the significance of attending a secure and supportive school environment. Children who lack access to such an environment are more prone to experiencing overweight or obesity. This highlights the need for schools to prioritize creating safe and nurturing environments for their students [16]. Collaboration between educators, health professionals, policymakers, and parents is crucial in implementing interventions and programs that prioritize the health and well-being of students within the school context. By addressing issues such as promoting physical activity, and providing school sports facilities, schools can play a vital role in combating childhood overweight and obesity [10]. Continued research in this field is essential to gain insights into effective strategies to promote healthy behaviors and address childhood overweight and obesity within schools. By examining school-level characteristics and their impact on student health, we can identify key areas for intervention and develop evidence-based approaches. Extending these findings beyond Pakistan and considering their applicability in other countries is crucial. While cultural and contextual factors may differ, the principles of creating supportive school environments and prioritizing children’s health are universal. This study provides a valuable foundation for informing practical actions and guiding programming, both within Pakistan and in other nations facing similar challenges. Engaging all stakeholders, including educators, health professionals, policymakers, and parents, in collaborative efforts is necessary to implement comprehensive school-based interventions. By working together, we can create environments where children develop healthy habits, reducing the risk of overweight and obesity and setting them on a path towards a healthier future.

The strengths of the study include the following. This is the first study of its kind, providing data on school sports facilities in Pakistan, where no such data previously existed. The current study significantly contributes to understanding Pakistan’s health challenges and global health trends by providing actual data on school-level factors and school sports facility parameters among Pakistani school children and adolescents. It illuminates the association between school-level physical activity, physical education, and body weight issues. Drawing comparisons with existing theories and findings underscores the comprehensiveness of our analysis, especially when considering demographic characteristics. Unlike previous studies limited to specific age groups or locations, our sampling technique ensured a broader representation of Pakistan’s diverse population. Additionally, this study is the first to comprehensively explore the relationship between weight status and various physical activity and physical education factors in Pakistan. Despite some nonsignificant findings, this study highlights the need for nuanced analyses considering interactions between physical activity, physical education variables, and BMI categories. Moving forward, interventions and policies aimed at addressing overweight and obesity in Pakistan and beyond can leverage the foundational data provided by this study. By focusing on an underrepresented population, this research fills critical gaps, providing updated insights into the prevalence and associations of overweight and obesity among Pakistani children and adolescents.

The current study had several major limitations. Firstly, its cross-sectional design precludes the inference of causality for the observed associations. Moreover, reliance on self-reported data, including information on diet-related behaviors and anthropometric measurements, introduces the possibility of information bias. Additionally, the study’s focus solely on school-aged children and adolescents aged 9 to 17 years limits the generalizability of findings to the broader Pakistani population. Exclusion of younger age groups (grades 1 through 3) may overlook critical factors related to overweight and obesity during primary school years. Furthermore, the reluctance of some girls’ school principals to permit measurements for girls aged 12 to 17 years affected the representation of girls in the sample. Notably, overweight and obesity were determined solely based on BMI calculations using the reference BMI chart, overlooking the assessment of body fat percentage, which could offer a more comprehensive measure of adiposity. These limitations underscore the need for cautious interpretation of our findings and suggest avenues for future research to address these gaps.

## 5. Conclusions

In conclusion, the prevalence of overweight and obesity among school-aged children and adolescents in Pakistan has increased significantly over recent decades. This study reveals substantial gaps in promoting physical activity and exercise within school environments, with a notable prevalence of zero sessions per week for physical education activities, morning exercises, and classroom exercises. There are also significant deficiencies in the availability of physical education teachers, physical activity programs, and sports facilities at the primary and middle school levels. The lack of adequate sports equipment and facilities to meet the requirements of daily physical education is significantly associated with students’ body weight status. These findings underscore the urgent need for interventions to address these issues and enhance physical education resources, standardized testing processes, and provisions for sports injury insurance and physical education support in schools. Furthermore, the comparison of body weight status prevalence with school-level parameters highlights associations between specific factors, such as leadership attitudes, provision of sports facilities, and playground satisfaction, and lower risks of overweight and obesity. Addressing these factors could contribute to creating a healthier school environment and potentially reducing the prevalence of overweight and obesity among students.

Based on the results, it is crucial to develop targeted interventions, especially for primary and middle schools lacking physical education teachers (PETs) and where physical activity is minimal. These interventions should aim to enhance the health of students in Pakistan. To assess their effectiveness, schools should conduct annual anthropometric measurements and implement fatness tests at school sports facilities. Future research should employ longitudinal or interventional studies to explore the relationship between overweight, obesity, fatness tests, and other health indicators. Continued research and targeted interventions are essential to fostering lifelong physical activity habits and improving student health and well-being.

## Figures and Tables

**Table 1:** Characteristics of school demographics.

Characteristics	*n* (%)
Sex	
Boy	2437 (59.3)
Girl	1671 (40.7)
Religion	
Muslim	3952 (96.2)
Christian	156 (3.8)
Student Residence	
Urban	2460 (59.9)
Rural	1648 (40.1)
School Location	
Urban	33 (53.2)
Rural	29 (46.8)
School Type	
Public	45 (72.6)
Private	17 (27.4)
School Level	
Primary specific	4 (6.5)
Primary section + Middle classes	3 (4.8)
Primary + Middle + Secondary classes	38 (61.3)
Secondary + Higher Secondary classes	17 (27.4)
Total	62 100.0

**Table 2:** Chi-square test comparing school type with prevalence of fatness-related situations among school-level factors.

		Primary School	Middle School	Secondary School	Higher Secondary School		
Characteristics	Type	*n* (%)	*n* (%)	*n* (%)	*n* (%)	*χ* ^2^	*p*-Value
Reasons for the failure to carry out physical education courses as planned over the past year.							
Weather, Mid-Final Exam	Public	2 (7.7)	1 (3.8)	15 (57.7)	8 (30.8)	3.27	0.351
	Private	0 (0.0)	0 (0.0)	5 (100.0)	0 (0.0)		
Other	Public	2 (10.5)	0 (0.0)	10 (52.6)	7 (36.8)	5.71	0.127
	Private	0 (0.0)	2 (16.7)	8 (66.7)	2 (16.7)		
Statistics of school fitness testing equipment.							
Excellent	Public	0 (0.0)	0 (0.0)	2 (33.3)	4 (66.7)	2.39	0.122
	Private	0 (0.0)	0 (0.0)	4 (80.0)	1 (20.0)		
Poor	Public	4 (10.3)	1 (2.6)	23 (59.0)	11 (28.2)	6.24	0.100
	Private	0 (0.0)	2 (16.7)	9 (75.0)	1 (8.3)		
The degree of standardization of the student physical fitness testing process.							
Extraordinary Specification	Public	0 (0.0)	0 (0.0)	0 (0.0)	1 (100.0)	5.00	0.082
	Private	0 (0.0)	1 (25.0)	3 (75.0)	0 (0.0)		
Non-Specification	Public	4 (9.1)	1 (2.3)	25 (56.8)	14 (31.8)	3.64	0.302
	Private	0 (0.0)	1 (7.7)	10 (76.9)	2 (15.4)		
School’s statistics on the accuracy of the assessment of students’ most recent physical fitness test data.							
Very accurate	Public	1 (20.0)	0 (0.0)	1 (20.0)	3 (60.0)	5.15	0.161
	Private	0 (0.0)	1 (33.3)	2 (66.7)	0 (0.0)		
Very inaccurate	Public	3 (7.5)	1 (2.5)	24 (60.0)	12 (30.0)	3.19	0.363
	Private	0 (0.0)	1 (7.1)	11 (78.6)	2 (14.3)		
Purchase of sports injury insurance for students in schools.							
Yes	Public	0 (0.0)	0 (0.0)	4 (50.0)	4 (50.0)	4.55	0.103
	Private	0 (0.0)	1 (20.0)	4 (80.0)	0 (0.0)		
No	Public	4 (10.8)	1 (2.7)	21 (56.8)	11 (29.7)	3.07	0.380
	Private	0 (0.0)	1 (8.3)	9 (75.0)	2 (16.7)		
Other departments or agencies are paired to support physical education in schools.							
Yes	Public	0 (0.0)	0 (0.0)	3 (50.0)	3 (50.0)	7.00	0.030
	Private	0 (0.0)	1 (100.0)	0 (0.0)	0 (0.0)		
No	Public	4 (10.3)	1 (2.6)	22 (56.4)	12 (30.8)	4.65	0.199
	Private	0 (0.0)	1 (6.3)	13 (81.3)	2 (12.5)		
Total		4 (6.5)	3 (4.8)	38 (61.3)	17 (27.4)		

**Table 3:** Chi-square test assessing the association of school sports facility parameters with body weight status by school type-specific trends among children and adolescents.

			Body Weight Status				
		Underweight	Healthy	Overweight	Obesity		
Characteristics	Type	*n* (%)	*n* (%)	*n* (%)	*n* (%)	*χ* ^2^	*p*-Value
A description of the attitude of school sports staff towards physical education in schools.							
Excellent	Public	140 (18.4)	431 (56.6)	131 (17.2)	59 (7.8)	3.48	0.323
	Private	99 (17.5)	302 (53.5)	106 (18.8)	58 (10.3)		
Poor	Public	371 (16.7)	1161 (52.3)	439 (19.8)	248 (11.2)	4.48	0.214
	Private	81 (14.4)	285 (50.6)	121 (21.5)	76 (13.5)		
The school provides students with access to sports facilities.							
Yes	Public	396 (17.6)	1211 (53.9)	416 (18.5)	224 (10.0)	4.38	0.230
	Private	128 (16.1)	415 (52.3)	152 (19.2)	98 (12.4)		
No	Public	115 (15.7)	381 (52.0)	154 (21.0)	83 (11.3)	1.13	0.961
	Private	52 (15.5)	172 (51.3)	75 (22.4)	36 (10.7)		
Survey of children and adolescents’ satisfaction with playgrounds.							
Yes	Public	335 (17.7)	1040 (54.8)	335 (17.7)	187 (9.9)	2.09	0.554
	Private	139 (16.8)	449 (54.4)	163 (19.8)	74 (9.0)		
No	Public	176 (16.3)	552 (51.0)	235 (21.7)	120 (11.1)	16.42	0.001
	Private	41 (13.5)	138 (45.5)	64 (21.1)	60 (19.8)		
Physical education teachers available in school.							
Yes	Public	409 (18.0)	1246 (54.9)	415 (18.3)	198 (8.7)	4.91	0.786
	Private	111 (16.1)	379 (54.9)	135 (19.6)	65 (9.4)		
No	Public	102 (14.3)	346 (48.6)	155 (21.8)	109 (15.3)	1.06	0.234
	Private	69 (15.8)	208 (47.5)	92 (21.0)	69 (15.8)		
Analysis of the effective use of funds for physical education in schools.							
Yes	Public	268 (17.6)	831 (54.5)	273 (17.9)	152 (10.0)	4.70	0.194
	Private	71 (15.3)	246 (53.0)	90 (19.4)	57 (12.3)		
No	Public	243 (16.7)	761 (52.3)	297 (20.4)	155 (10.6)	0.20	0.976
	Private	109 (16.4)	341 (51.4)	137 (20.6)	77 (11.6)		
The condition of the school’s sports equipment facilities to meet the requirements of daily PE.							
Yes	Public	88 (18.9)	256 (55.1)	83 (17.8)	38 (8.2)	10.88	0.012
	Private	29 (14.6)	103 (51.8)	39 (19.6)	28 (14.1)		
No	Public	423 (16.8)	1336 (53.1)	487 (19.4)	269 (10.7)	0.22	0.973
	Private	151 (16.3)	484 (52.1)	188 (20.2)	106 (11.4)		
The school sports venue is open to students free of charge on weekends.							
Yes	Public	88 (18.9)	256 (55.1)	83 (17.8)	38 (8.2)	6.88	0.074
	Private	29 (14.6)	103 (51.8)	39 (19.6)	28 (14.1)		
No	Public	423 (16.8)	1336 (53.1)	487 (19.4)	269 (10.7)	1.50	0.680
	Private	151 (16.3)	484 (52.1)	188 (20.2)	106 (11.4)		
Total		691 (16.8)	2179 (53.0)	797 (19.4)	441 (10.7)		

**Table 4:** Correlation between school-level factors and body weight status.

Characteristics		1	2	3	4	5	6	7
1	Body weight status	—						
2	Leadership of attitudes towards school sports.	0.053 **	—					
3	Provide facilities for student use of sports venues.	0.049 **	0.095 **	—				
4	Students’ satisfaction with the playground.	0.065 **	0.169 **	0.676 **	—			
5	Effective use of funds for physical education in schools.	0.021	0.262 **	0.193 **	0.379 **	—		
6	School sports facilities meet daily physical education requirements.	0.018	0.333 **	0.241 **	0.277 **	0.453 **	—	
7	The school sports grounds are free for students to use on weekends.	0.035 *	0.178 **	0.204 **	0.229 **	0.147 **	0.032 *	—

Note: *N* = 4108; * *p* < 0.05, ** *p* < 0.01.

**Table 5:** Linear regression analysis of school-level school sports facility parameters and body weight status.

		Unstandardized Coefficients		Standardized Coefficients		
	Characteristics	B	SE	*β*	*t*	Sig.
	Constant	1.943	0.106		18.378	<0.001
1	Leadership of attitudes towards school sports.	0.086	0.031	0.047	2.767	0.006
2	Provide facilities for student use of sports venues.	0.019	0.042	0.010	0.448	0.654
3	Students’ satisfaction with the playground.	0.100	0.041	0.055	2.437	0.015
4	Effective use of funds for physical education in schools.	−0.020	0.032	−0.012	−0.621	0.535
5	School sports facilities meet daily physical education requirements.	−0.025	0.043	−0.011	−0.589	0.556
6	The school sports grounds are free for students to use on weekends.	0.038	0.045	0.014	0.850	0.395

SE = Standard error.

**Table 6:** Odds ratios from two logistic regression analyses of school-level factors and risk of overweight and obesity.

	Overweight vs. Non-Overweight	Obese vs. Non-Obese
Characteristics	Unadjusted OR (95% CI)	Unadjusted OR (95% CI)
Leadership attitudes towards school sports.		
Excellent	0.86 (0.73–1.02)	0.73 (0.58–0.91) **
Poor	Ref.	Ref.
Provide facilities for student use of sports venues.		
Yes	0.86 (0.73–1.03)	0.76 (0.61–0.95) *
No	Ref.	Ref.
Satisfaction with the playground.		
Yes	0.81 (0.69–0.95) *	0.71 (0.58–0.87) **
No	Ref.	Ref.
Effective use of funds for physical education.		
Yes	0.86 (0.74–1.01)	0.95 (0.78–1.16)
No	Ref.	Ref.
Facilities meet requirements for daily physical education.		
Yes	0.92 (0.74–1.14)	0.90 (0.68–1.19)
No	Ref.	Ref.
Sports grounds are free to open on weekends.		
Yes	0.97 (0.76–1.25)	0.76 (0.51–1.08)
No	Ref.	Ref.

Level of significance: * *p* < 0.05, ** *p* < 0.01. CI = Confidence Interval, OR = Odds Ratio. Note: Reference category (respectively): Ref.

## Data Availability

The corresponding author can provide the data used in this work upon request.
